# Metastatic Breast Cancer Prevalence in New South Wales, Australia, in 2016: A Health Record Linkage Study

**DOI:** 10.1111/ajco.14176

**Published:** 2025-04-29

**Authors:** Andrea L. Smith, Xue Qin Yu, Dianne L. O'Connell, Nehmat Houssami, Belinda E. Kiely, Anne E. Cust, David P. Smith, Michael David, Sarah J. Lord

**Affiliations:** ^1^ The Daffodil Centre University of Sydney A Joint Venture with Cancer Council NSW Sydney New South Wales Australia; ^2^ Wiser Healthcare School of Public Health Faculty of Medicine and Health University of Sydney Sydney New South Wales Australia; ^3^ NHMRC Clinical Trials Centre University of Sydney Sydney New South Wales Australia; ^4^ School of Medicine and Dentistry Griffith University Gold Coast Queensland Australia

**Keywords:** cancer registries, epidemiology, health record linkage, metastatic breast cancer, prevalence

## Abstract

**Aim:**

To estimate the number of females living with metastatic breast cancer (MBC) in New South Wales (NSW), Australia, in 2016 using linked health records.

**Methods:**

The primary study dataset (cohort 1) included females in the NSW Cancer Registry (NSWCR) with breast cancer diagnosed during 2001–2002 and 2006–2007 linked with administrative hospital records, medicine dispensing, radiation services, and death records. From this dataset we counted the number with a record of de novo MBC or recurrent MBC (following stage I–III cancer) alive at the end of each year (2001–2015). The second dataset (cohort 2) included females with breast cancer diagnosed 2003–2005 and 2008–2015 without linked records. We imputed MBC prevalence for cohort 2 by calculating MBC prevalence proportions at the end of each year in cohort 1 and applying these proportions to NSWCR incidence counts in cohort 2.

**Results:**

Cohort 1 comprised 16,521 females with breast cancer, of whom 4364 had MBC recorded (976 de novo; 3388 recurrent). A total of 1245 individuals with MBC recorded were alive on January 1, 2016 (270 de novo, 21.7%; 975 recurrent, 78.3%). When extrapolated to all females diagnosed with breast cancer in 2001–2015 in NSW, 5009 individuals were estimated to be living with MBC on January 1, 2016 (1609 de novo, 32.1%; 3400 recurrent, 67.9%).

**Conclusion:**

This study estimates that a large number of individuals are living with MBC and demonstrates the importance of identifying individuals with recurrent MBC, in addition to de novo MBC, to inform funding and delivery of appropriate clinical and supportive care services.

## Graphical abstract

This study demonstrates the feasibility of using linked health records to estimate metastatic breast cancer prevalence. It demonstrates the importance of identifying individuals with recurrent metastatic breast cancer, in addition to de novo metastatic breast cancer, to inform funding and delivery of appropriate clinical and supportive care services

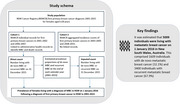

## 1 Introduction

Breast cancer is the most commonly diagnosed cancer worldwide. In 2020, 2.3 million people were diagnosed with breast cancer and there were 685,000 deaths, representing 1 in every 6 cancer deaths in females [1]. In high‐income countries such as Australia, almost all breast cancer deaths are in people with metastatic disease. Until 2000, the median survival for people diagnosed with metastatic breast cancer (MBC) was less than 2 years [2]. Since then, treatment advances have extended post‐metastasis survival for many patients [3, 4], and MBC is now frequently referred to as a treatable but not curable cancer [5], with median survival times closer to 5 years [6, 7]. However, the number of people living with MBC is not known. Although population‐based cancer registries may report the number of people with metastatic cancer at initial cancer diagnosis (‘de novo’ MBC), the number of people with cancer recurrence after an initial diagnosis of non‐metastatic disease (referred to herein as ‘recurrent’ MBC) is not routinely reported, which makes it difficult to estimate post‐metastasis survival for those with recurrent MBC. Given that it has been estimated that people diagnosed with recurrent MBC comprise about two‐thirds of the MBC population, and their clinical and supportive care needs are similar to those diagnosed with de novo MBC, it is important that they are included in prevalence estimates [8, 9].

The lack of data on metastatic cancer prevalence is a major evidence gap. This information is essential for planning and delivering appropriate cancer services for this population. Cancer services required by those with MBC include surveillance for cancer progression (scans, blood tests, and other diagnostics), treatment (systemic therapies, radiotherapy, and surgery), supportive care (management of symptoms and side effects; psychosocial support; practical care including financial advice relating to employment, superannuation/pensions and life insurance), and end‐of‐life care. As treatment aims to slow the growth and spread of the cancer and reduce cancer symptoms for as long as possible, patients require access to these services on an ongoing basis. Costs associated with MBC (medical and productivity) are higher than for early breast cancer and in the United States have been projected to increase by 140% between 2015 and 2030 [10, 11].

In the absence of cancer registration and counts of MBC in Australia, the advocacy organization Breast Cancer Network Australia estimated the prevalence of MBC in 2020 to be 10,553 by applying Clements and colleagues’ modeled ratio of prevalent cases to the number of breast cancer deaths by age group reported for Australian females aged 18–84 years in 2020 [12, 13]. Given the improvements in survival for MBC since the period used in the modeling (1980–2004) [3, 4], this estimate is likely to be conservative. Thus, in this study, we proposed using data from the New South Wales (NSW) Cancer Registry (2001–2002, 2006–2007) linked to administrative hospital records, medicine dispensing, radiation services, and death records to estimate MBC prevalence.

This study aimed to estimate the number of females living with de novo or recurrent MBC in NSW January 1, 2016 following a diagnosis of breast cancer during the previous 15 years, 2001 to 2015.

## 2 Methods

### 2.1 Data Sources

We used two population‐based datasets from the NSW Cancer Registry (NSWCR). The primary dataset (cohort 1) comprised individual records of first primary female breast cancers diagnosed in 2001–2002 and 2006–2007 linked with administrative health records [14]. The secondary dataset without linked health records (cohort 2) comprised aggregated incidence counts of first primary female breast cancers diagnosed in the years that linked data were not available (2003–2005 and 2008–2015). These counts were aggregated by year of diagnosis, 10‐year age group, and disease stage at diagnosis (localized, regional, distant, and unknown).

### 2.2 Record Linkage

**FIGURE 1:**
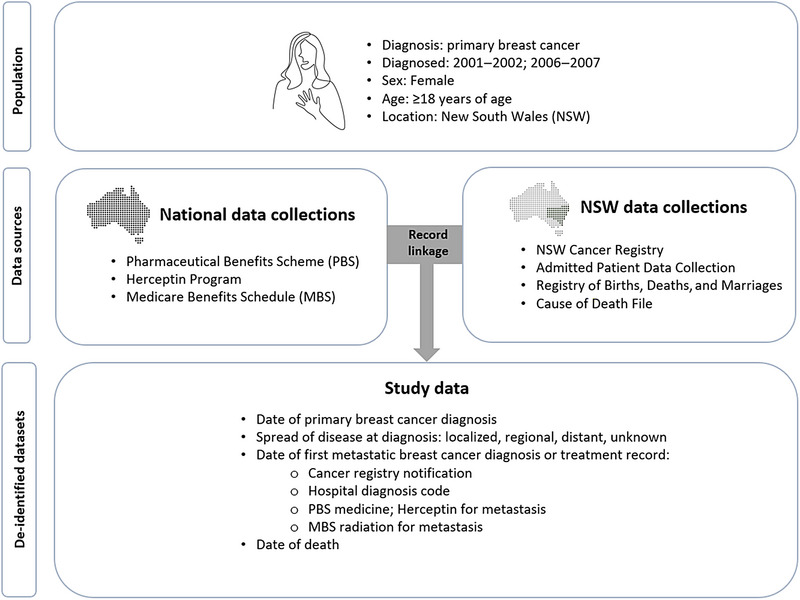
Population, data sources, record linkages, and study data for the record linkage dataset (cohort 1).

Cohort 1 included all females aged ≥18 years with a diagnosis of first primary breast cancer registered in the NSWCR for 4 years: 2001–2002 and 2006–2007. All females meeting these criteria were included, including those who also had non‐breast primary cancers registered in the NSWCR at any time. Males were excluded. Data sources for linked records were the NSWCR, NSW Registry of Births, Deaths and Marriages (RBDM), the NSW Admitted Patient Data Collection (APDC) for hospital records, Commonwealth Government Pharmaceutical Benefits Scheme (PBS) for dispensed prescription medicines, and Medicare Benefits Schedule (MBS) claims for government subsidized radiotherapy services (Figure 1). The details of these datasets and linkage have been previously reported [14]. We used NSWCR records to stratify by 10‐year age group, extent of spread at diagnosis (localized, regional, distant, and unknown).

### 2.3 Outcomes

The primary study outcomes were de novo MBC, recurrent MBC following an initial diagnosis of localized, regional, or unknown stage breast cancer, and death from any cause. We defined de novo MBC as an NSWCR record of distant disease at diagnosis. The NSWCR defines distant disease at diagnosis using the Degree of Spread classification from the International Agency for Research on Cancer. This definition classifies people as having distant disease based on notifications received within 120 days of the date of breast cancer diagnosis. To classify recurrent MBC, we used the definition of distant metastasis from the UICC/ American Joint Committee on Cancer (AJCC) TNM classification system (8th edition) [15].

To identify MBC in cohort 1, we used a six‐criteria definition of MBC developed for Australian administrative health records (Supplementary file) [16]. Of the six criteria, five were used to estimate the date of first recurrent MBC diagnosis for each person with non‐metastatic BC in cohort 1 from January 1, 2001 to December 31, 2015. We determined the date of death from the NSWCR and RBDM (January 1, 2001 to December 31, 2015) to classify each person with an MBC record as alive or dead on January 1, 2016.

### 2.4 Analysis

**FIGURE 2:**
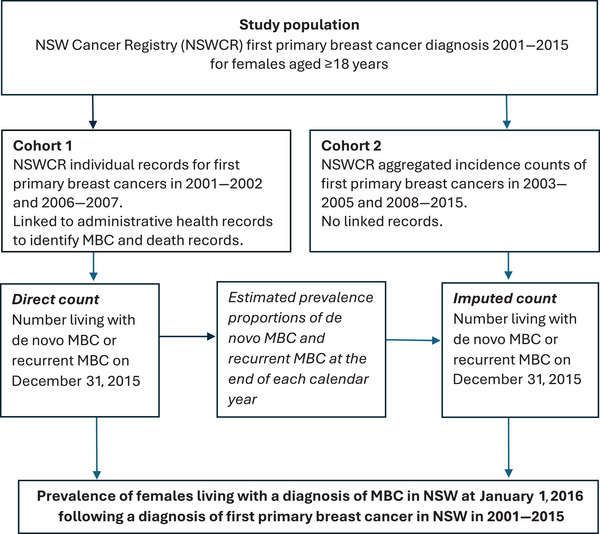
Study design schema. MBC: metastatic breast cancer; NSW: New South Wales; NSWCR: New South Wales Cancer Registry.

We used the direct counting method to estimate 15‐year MBC prevalence on January 1, 2016 [17]. This analysis involved three principal activities. First, using cohort 1, we identified individuals with de novo or recurrent MBC and calculated prevalence proportions on 31 December of each calendar year (2001–2015) separately for de novo MBC and recurrent MBC. For de novo MBC cases, the proportion was the number of de novo MBC alive at the end of a given year divided by the number of de novo MBC cases. Similarly, the prevalence proportion was calculated for recurrent MBC cases. Then, in cohort 2, we imputed MBC prevalence for the years in which linked data were not available (2003–2005 and 2008–2015) by applying the prevalence proportions calculated from cohort 1 to the aggregated breast cancer incidence data. This imputation approach assumes that the risk of recurrent MBC and post‐metastasis survival remained constant for the years in which linked data were not available. This is a basic assumption that might under‐ or overestimate the prevalence of MBC as more effective neoadjuvant and adjuvant therapies to reduce the risk of recurrent MBC and systemic therapies to extend survival for metastatic disease have been introduced over time. Finally, we counted all individuals living with MBC on December 31, 2015, combining the observed prevalence for 2001–2002 and 2006–2007 from cohort 1 and imputed prevalence for cohort 2 (Figure 2).

Age and extent of disease at diagnosis are recognized as important risk factors for recurrent MBC and therefore MBC prevalence [16, 18].  Age is also an important prognostic factor for MBC survival [16, 19].  We therefore performed a sensitivity analysis for the imputation of recurrent MBC from non‐metastatic breast cancer in cohort 1 with stratification of counts by age at diagnosis (<50, 50–69, and ≥70 years) or extent of disease (localized, regional, and unknown) to take account of temporal changes in their distribution.

## 3 Results

The NSWCR registered 68,999 first primary breast cancers in females aged ≥18 years in NSW during 2001–2015. Of these, 16,521 (24%) were included in cohort 1 (record linkage data available) (Table 1). The distribution of age at breast cancer diagnosis was similar between cohorts 1 and 2 (Table 1). A total of 976 females with de novo MBC were recorded in cohort 1 (5.9%) and 3228 (6.2%) in cohort 2. The main difference in the spread of disease at diagnosis over the study period was a lower proportion of cancers recorded as unknown stage at diagnosis over time (2001–2002 9.3%, 2008–2015 5.8%). Cohort 1 included 1344 (8%) females with cancer registry records of a prior primary non‐breast cancer in addition to breast cancer (data not available for cohort 2).

**TABLE 1:** Characteristics of NSW females aged ≥18 years with a first primary invasive breast cancer diagnosis in the record linkage cohort (cohort 1; diagnosed 2001–2002, 2006–2007) and those without linked records (cohort 2; diagnosed 2003–2005 or 2008–2015).

	All breast cancer diagnoses	Cohort 1 (linked records)	Cohort 2 (no linked records)	Cohort 1 (linked records)	Cohort 2 (no linked records)
Diagnosis period of initial breast cancer	2001–2015	2001–2002	2003–2005	2006–2007	2008–2015
			*N* (%)		
All	68,999	8132 (11.8)	12,304 (17.8)	8389 (12.2)	40,174 (58.2)
Extent of disease at diagnosis					
Localized	35,402 (51.3)	4246 (52.2)	6280 (51.0)	4242 (50.6)	20,634 (51.4)
Regional	24,802 (35.9)	2659 (32.7)	4323 (35.1)	3146 (37.5)	14,674 (36.5)
Distant	4204 (6.1)	467 (5.7)	681 (5.5)	509 (6.1)	2547 (6.3)
Unknown	4591 (6.7)	760 (9.3)	1020 (8.3)	492 (5.9)	2319 (5.8)
Age at breast cancer diagnosis, years					
<50	15,635 (22.7)	1918 (23.6)	3018 (24.5)	1978 (23.6)	8721 (21.7)
50–69	34,609 (50.2)	3904 (48.0)	6002 (48.8)	4328 (51.6)	20,375 (50.7)
≥70	18,755 (27.2)	2310 (28.4)	3284 (26.7)	2083 (24.8)	11,078 (27.6)

### 3.1 MBC Prevalence

We estimated the 15‐year prevalence of MBC in NSW on January 1, 2016 was 5009, comprising 1609 (32%) prevalent de novo metastasis and 3400 (68%) recurrent MBC from our direct counts (cohort 1) and imputations (cohort 2) (Table 2).

**TABLE 2:** Prevalence of MBC on January 1, 2016, direct counts for the record linkage cohort (cohort 1; diagnosed 2001–2002, 2006–2007) and imputed for those without linked records (cohort 2; diagnosed 2003–2005 or 2008–2015).

	All	Cohort 1 (direct counts)	Cohort 2 (imputed)	Cohort 1 (direct counts)	Cohort 2 (imputed)
Diagnosis period of first primary breast cancer	2001–2015	2001–2002	2003–2005	2006–2007	2008–2015
Incident breast cancer	68,999	8132	12,304	8389	40,174
Incident MBC counts					
De novo MBC	4204	467	681	509	2547
Recurrent MBC	NA	1982	NA	1406	NA
Prevalent MBC on January 1, 2016, *N* (%)					
De novo MBC	1609 (32)	133 (21)	194 (22)	137 (22)	1145 (40)
Recurrent MBC	3400 (68)	492 (79)	689 (78)	483 (78)	1736 (60)
All	5009	625	883	620	2881

Abbreviations: MBC, metastatic breast cancer; NA, not available.

The direct prevalence count of MBC for the 2001–2002 and 2006–2007 cohort 1 was 1245, comprising 270 (22%) prevalent de novo metastasis and 975 (78%) recurrent MBC. The imputed prevalence count of MBC for cohort 2 was 3764, comprising 1339 (36%) prevalent de novo metastasis and 2425 (64%) recurrent MBC (Table 2). The proportion of recurrent MBC was lower for cohort 2, reflecting the relatively lower incidence of recurrent MBC in the 2008–2015 group who had a shorter period from their initial breast cancer diagnosis to experience recurrence. Results from the sensitivity analysis for imputation of recurrent MBC produced very close estimates (3407 by age at diagnosis and 3404 by extent of disease).

## 4 Discussion

Our study responds to Australian and international cancer policy and consumer advocacy recommendations for research to generate evidence‐based estimates of the number of people living with MBC [12, 20–23]. To our knowledge, this is the first Australian study to demonstrate the feasibility of using linked health records to estimate MBC prevalence to address this important cancer data gap. We used direct counting and imputation to estimate that 5009 females with a diagnosis of MBC were living in NSW at the beginning of 2016. Of these, two‐thirds had metastatic disease diagnosed after recurrence of earlier stage breast cancer. For context, in the prior year (2015), NSW recorded 290 new diagnoses of de novo MBC and 5372 new diagnoses of breast cancer without distant metastases in females, and 37 new diagnoses of breast cancer in males. Together, these statistics indicate that, although only about 5% of new breast cancers have distant metastatic spread at diagnosis, the total number of individuals living with and being treated for MBC is nearly as high as the number undergoing treatment each year for an initial early‐stage breast cancer diagnosis.

Extrapolating from our study findings in NSW, we estimate that approximately 15,000 individuals in Australia were living with a diagnosis of MBC at the beginning of 2016. This figure was calculated by applying a factor of three to our study results, representing the ratio of total breast cancers diagnosed in Australia each year compared to NSW alone, and assuming de novo and recurrent MBC incidence and survival rates observed in NSW are similar across Australia. This estimate is considerably larger than previous Australian modeled estimates for 2004 from Clements and colleagues (*n* = 8284) and for 2020 (*n* = 10,553) from Breast Cancer Network Australia using Clements and colleagues’ modeling methods [12, 21].

Given the improvements in survival for MBC since 2004, and the growing and aging population leading to more breast cancer diagnoses each year, it is not unexpected that the number of people living with MBC is increasing. This concurs with what has been reported in the United States for metastatic cancers in general and MBC specifically. In 2022, Gallicchio and colleagues used the Mortality and Incidence Analysis Model (MIAMOD) approach to estimate the number of metastatic cancer survivors for the most common cancers in the United States (breast, prostate, bladder, lung, melanoma, and colorectal). They reported a substantial increase in prevalence between 1990 and 2018 (623,405 estimated for 2018; 693,452 projected for 2025) [8]. For MBC, Gallicchio and colleagues reported that MBC prevalence increased linearly from 1990 to 2018. An estimated 140,230 women were living with MBC in the United States on January 1, 2018 and this is projected to increase to 169,347 by 2025 [8]. They also reported that one‐fifth of individuals diagnosed with MBC were estimated to have been living with MBC for 10 years or more.

Gallicchio and colleagues’ findings indicate a relative increase of 55% in MBC prevalence between 2005 and 2025, suggesting the true prevalence of MBC in Australia in 2024 may be higher than our present estimate for 2016. One limitation of the US analysis is that, in the absence of US population‐level data on post‐metastasis survival for recurrent MBC, the model applied a 1.35 higher risk of cancer death for recurrent MBC relative to de novo MBC survival based on a single institution study of recurrent MBC diagnosed 1992–2007. The investigators noted that prevalence would be “markedly higher” if post‐metastasis survival for recurrent MBC was assumed to be similar to that for de novo MBC [8].

Using record linkage and direct counts, White et al. in 2021 estimated the prevalence of metastatic cancer and the number of people living in England at the end of 2015 with a treatable but not curable cancer [5]. They defined treatable but not curable cancer as a cancer that is highly unlikely to be eradicated and that, in the absence of other more imminent causes of death, is highly likely to lead to death. They estimated that at the end of 2015, 162,561 people were living with treatable but not curable cancer of whom 12,209 had treatable but not curable MBC. This number excluded individuals who died in the following year and were categorized as having end‐of‐life MBC.

White et al. had access to linked data for all individuals with a new diagnosis of breast cancer recorded during 2001–2015, thus did not need to include imputation. Nevertheless, the study also illustrated the inherent challenges of using routinely collected administrative data to identify MBC. Linked data were only available in England for the years 2012–2015 which did not allow identification of individuals with a diagnosis of recurrent MBC who completed treatment before 2012 and were alive at the end of 2015. Further, similar to the present study, the investigators noted the challenges of identifying distant recurrences from medicine dispensing records because some medicines’ indications were not restricted to metastatic disease and these indications may change over time.

### 4.1 Limitations

The major limitation of our record linkage dataset is the potential for over‐ or underdetection of recurrent MBC from administrative records which may result in over‐ or underestimation of prevalence. The highest potential for misclassification may be from medicine dispensing records due to the lack of staging information. For example, to distinguish treatment of distant recurrence from locoregional recurrence, we relied on locoregional recurrence triggering a hospital record (e.g., surgery) or radiotherapy record, which may have led to some misclassification of locoregional recurrence as distant recurrence (‘false positive’ MBC). Conversely, people with a distant recurrence who were treated with endocrine therapy alone without triggering a cancer registry, hospital, or radiotherapy record would not be detected using the study MBC criteria (‘false negative’ MBC).

We found 8% of the record linkage cohort had another primary cancer recorded, our estimate of MBC prevalence may include metastatic disease due to other primaries. However, we consider these people (who are typically excluded from population‐based studies of recurrent MBC) are important to include in prevalence estimates given the support service needs are likely to be similar across cancers. Internationally, cancer registry studies using administrative records to identify MBC have reported sensitivity and specificity of up to 97% for detection of breast cancer recurrence [24]. However, these studies were designed to validate record linkage methods for estimating the incidence of breast cancer recurrence and have generally been conducted over relatively short follow‐up periods. The cumulative impact of false positive MBC records may be larger when using record linkage to estimate MBC prevalence.

The main limitation of the data imputation approach is that the derived estimates of MBC prevalence proportions were restricted to the outcomes observed in the record linkage cohort which included breast cancer diagnoses in 2001–2002 and 2006–2007, but the risk of recurrent MBC and post‐metastasis survival and therefore MBC prevalence may change over time due to advances in diagnosis and treatment [3]. Thus, although this estimate applies to the year 2016, these cohort data nevertheless represent the best available data for Australia at present. Modeling approaches are similarly restricted to estimates from existing data, in particular post‐metastasis survival after recurrent MBC which is known to differ from that for de novo MBC and often drawn from more limited breast cancer cohorts [8].

### 4.2 Implications

The exact number of people living with MBC in Australia is unknown. Prevalence information is critical as a first step to understanding the burden of MBC in the Australian community and essential for healthcare planning and resource allocation. Internationally, there are increasing calls for cancer registries to collect recurrence data. The 2024 Lancet Breast Cancer Commission's roadmap for change stated that we should be aiming for a minimum of 70% of global cancer registries registering people with MBC [25]. These data are essential to allow direct counting of MBC incidence to improve the quality of MBC prevalence estimates and to allow ongoing surveillance. Record linkage provides an important interim approach until cancer registries are funded to collect and report these data. Modeling will continue to be a valuable complement to record linkage approaches, including in countries where record linkage is not feasible.

By demonstrating the feasibility of using linked health records to estimate MBC prevalence, our study supports extending record linkage to include complete 15–20 year linked datasets for cancer registry breast cancer cohorts (females and males) for all years, removing the need for imputation to provide a more robust estimate of the current prevalence of MBC in NSW and Australia. Including linked data from all years would also allow comparisons based on variables such as breast cancer subtype, place of residence or age to identify disparities in outcomes, an important focus in Australia's inaugural 10‐year Australian Cancer Plan [26]. In NSW, additional data sources relating to outpatient care are now available for linkage. Moreover, notifications to the cancer registry have improved since 2016 which supports cancer registry reporting of recurrence [27]. These methods can also be applied to other cancers [5].

In the future, additional benefits could be gained by linking cancer registries’ stage and recurrence data to treatment outcome and patient reported outcome measure (PROMs) datasets across the disease trajectory. Such linkages would allow policymakers and those in program delivery to understand better the burden of MBC and the impact that population‐wide programs such as screening, clinical care, treatment, and supportive care have on cancer recurrence and survival.

### 4.3 Future Research

To support the use of record linkage to identify metastatic cancer, there is a need to create common definitions and rules to harmonize this research across jurisdictions in Australia and between countries. Further research is needed to validate the Australian record linkage algorithm against high‐quality clinical data to estimate the accuracy of prevalence estimates generated through linked datasets. Validation studies are also needed to support refinement of the algorithm, identification of key limitations to aid interpretation, and strategies to improve the completeness and quality of recurrence notifications to the cancer registries.

To develop an understanding of this underresearched population, research priorities include population‐level information on MBC incidence (new cases per year) and prevalence, sites of first and subsequent recurrence, and post‐metastasis survival. Monitoring is required to assess the effectiveness of service delivery and reduced mortality by place of residence. Further research is also needed to define and measure important subgroups and trajectories relevant for delivering appropriate support services, including the populations who are treatable but not curable and people receiving end‐of‐life care [28, 29].

## 5 Conclusions

Our study demonstrates how, in the absence of cancer registry recurrence data, existing resources such as episode data from administrative health datasets can be leveraged to estimate MBC prevalence and provide data on the characteristics of people with MBC. These outputs can be used to inform the funding and delivery of appropriate clinical and supportive care services. Our findings demonstrate the importance of identifying people with both de novo and recurrent MBC and support the 2024 Lancet Breast Cancer Commission's call that all cancer registries should be collecting and reporting data on MBC, including stage at diagnosis and recurrence.

## Conflicts of Interest

The authors declare no conflicts of interest.

## Supporting information



Supporting Information
