# Supplementary material for: Metastatic Breast Cancer Prevalence in New South Wales, Australia, in 2016: A Health Record Linkage Study
Source: Asia Pac J Clin Oncol. 2025 Apr 29;21(4):407–14. doi: 10.1111/ajco.14176 (PMC12206281; doi:10.1111/ajco.14176)
Supplement: Supplementary file 1 — Supporting Information [file AJCO-21-407-s001.docx]

SUPPLEMENTARY FILE

Supplementary file: Criteria to identify metastatic breast cancer (MBC) from administrative health records

| **Data source** | **MBC criteria** |
| --- | --- |
| NSWCR | *Criterion 1.* De novo MBC recorded at initial breast cancer diagnosis; or for individuals with an initial diagnosis of non-metastatic breast cancer, a distant disease notification subsequently recorded in the NSWCR.  Notification of new and recurrent cancer cases to the NSWCR is a statutory requirement for hospitals, pathology laboratories, radiation therapy services, outpatient departments, day procedure centres and nursing homes. |
|  | MBC date was assigned as date of de novo MBC or first distant disease notification. |
| **Additional criteria 2-6 applied to identify recurrent MBC for individuals with an initial diagnosis of non-metastatic breast cancer** | |
| **Metastatic-specific** | |
| NSW APDC | *Criterion 2.* First hospital episode of care with ICD-10AM code for secondary malignant neoplasm as principle or secondary diagnosis (ICD 10-AM C77.1, C77.2, C77.4-C77.8, C78.0−C78.8, C79.0−C79.88).  Excludes ICD-10-AM codes for secondary cancer to lymph nodes that may indicate breast locoregional recurrence rather than distant spread: axilla/upper limb (C77.3); neck (C77.0) which includes supraclavicular nodes; and unspecified (C77, C77.9).  Distant recurrence date was assigned as first date of admission meeting this criterion. |
| MBS | *Criterion 3*. First radiation service with MBS code that specifies ‘secondary site’ (MBS item codes 15227, 15242, 15257, 15272).  These codes were introduced in May 2003, before this date, general radiation codes were used (see criterion 6).  Distant recurrence date was assigned as first date of radiation service meeting this criterion. |
| PBS | *Criterion 4.* Dispensing of anti-neoplastic medicine with PBS item code restricted to advanced/metastatic breast cancer.  ATC Codes: L01BC06, L01DB01, L01CD01, L01XC03 (advanced/metastatic item codes only), L01BC05, L01CA04, L01XX41, L01XE07, L01XC13, L01XC14, L01XE10, L01XE33, L01XE42, L02BA02, L02AB02, L02AB01.  Distant recurrence date was assigned as first date of dispensing meeting this criterion. |
| Herceptin Program | Trastuzumab prescription for treatment of metastatic breast cancer recorded by the Herceptin Program.  Trastuzumab was subsidised for this indication from December 2001 to June 2015 and from the PBS thereafter.  Distant recurrence date was assigned as first date of prescription meeting this criterion. |
| **Metastatic treatment pattern** | |
| PBS | *Criterion 5.* Dispensing of anti-neoplastic medicine indicated for treatment of metastatic breast cancer, but PBS item code not restricted to metastatic disease (ATC Codes: L01AA01, L01BC02, L01DB01, L01CD01, L01CD02, L01XA01, L01XA02, L01DB03), if meets the following criteria to distinguish from non-metastatic indications:  Treatment initiated after the initial adjuvant treatment period (adjuvant period defined as ≤12 months after the primary breast cancer registration date in the NSWCR); and after a treatment gap ≥90 days from prior adjuvant therapy, or treatment for locoregional recurrence or a second primary breast cancer. Locoregional recurrence or a second breast cancer primary were defined as: a second primary breast cancer record in the NSWCR; a medicine dispensing <90 days before or after hospital procedure record for breast or axillary lymph node surgery, or an MBS code for radiation that specified site as primary breast cancer.  Distant recurrence date was assigned as first date of dispensing meeting this criterion. |
| MBS | *Criterion 6.* Radiation service for palliative treatment, defined as <15 sequential (fractionated) services, but MBS code not restricted to secondary sites (MBS item codes 15203, 15204, 15207, 15208) and initiated after the initial adjuvant treatment period (adjuvant period defined as ≤12 months after the primary breast cancer registration date in the NSWCR).  Adjuvant radiation therapy defined as ≥25 sequential fractionated services, corresponding to standard practice at the time. Radiation services between these limits (16-24 fractionated services) were reviewed with radiation oncologist advice to classify as distant recurrence or not.  These general MBS codes were discontinued in April 2003, after this date, MBS radiation codes for secondary sites were used (see criterion 3).  Distant recurrence date was assigned as the first date of radiation service meeting this criterion. |

APDC = Admitted Patient Data Collection; NSWCR = New South Wales Cancer Registry; MBS = Medicare Benefits Schedule; PBS = Pharmaceutical Benefits Scheme
